# The application of JAK inhibitors in the peri-transplantation period of hematopoietic stem cell transplantation for myelofibrosis

**DOI:** 10.1007/s00277-024-05703-1

**Published:** 2024-03-18

**Authors:** Zerong Wang, Xuelian Jin, Jiajia Zeng, Zilin Xiong, Xinchuan Chen

**Affiliations:** West China Hospital, Sichuan University, Chendu, Sichuan China

**Keywords:** Myelofibrosis, JAK inhibitors, Ruxolitinib, Allogeneic hematopoietic stem cell transplantation, Poor implantation, Graft-versus-host disease

## Abstract

Myelofibrosis (MF) is a myeloproliferative neoplasm (MPN) with a poor prognosis, and allogeneic hematopoietic stem cell transplantation (allo-HSCT) is the only treatment with curative potential. Ruxolitinib, a JAK1/2 inhibitor, has shown promising results in improving patients’ symptoms, overall survival, and quality of life, and can be used as a bridging therapy to HSCT that increases the proportion of transplantable patients. However, the effect of this and similar drugs on HSCT outcomes is unknown, and the reports on their efficacy and safety in the peri-transplantation period vary widely in the published literature. This paper reviews clinical data related to the use of JAK inhibitors in the peri-implantation phase of hematopoietic stem cell transplantation for primary myelofibrosis and discusses their efficacy and safety.

## Introduction

Myelofibrosis (MF) can be divided into Primary MF (PMF) and Secondary MF (SMF) that evolves from polycythemia vera (PV) and essential thrombocythemia (ET). It is characterized by blood cell changes, systemic symptoms, splenic enlargement, extramedullary hematopoiesis, and collagen hyperplasia in the myeloid hematopoietic tissue. MF is Philadelphia chromosome-negative myeloproliferative neoplasm (MPN) with a poor prognosis. The estimated median survival is six years [1]. MF is usually associated with mutations in driver genes, mainly including the JAK2V617F mutation, MPL mutations, and CALR mutations. And the JAK2V617F mutation, in particular, is found in more than 95% of patients with PV and in about 50% of patients with ET and those with primary MF [2]. The JAK-STAT pathway is associated with the relevant signaling downstream to these mutations, and MF also frequently presents with cytokine storm-associated systemic inflammatory symptoms. In 2011, the JAK1/2 inhibitor ruxolitinib was approved in the United States for the treatment of medium- to high-risk MF. It has shown promising results in improving patients’ symptoms, overall survival and quality of life [3, 4]. But ruxolitinib (RUX) does not cure myelofibrosis or reduce the risk of acute leukemic transformation, and patients may even show clonal evolution due to the acquisition of new mutations. Therefore, the only treatment with curative potential remains allogeneic hematopoietic stem cell transplantation (allo-HSCT) [3, 5, 6]. MF patients undergoing allo-HSCT have a higher incidence of transplantation-related complications and mortality, especially implantation failure and graft-versus-host disease (GVHD). Significant efficacy has been observed with JAK inhibitors, represented by ruxolitinib (RUX), for the treatment of steroid-refractory GVHD. On the other hand, however, hematopoietic reconstitution and immune reconstitution after transplantation are also highly dependent on cytokines and the JAK-STAT signaling, and the use of JAK inhibitors carries the concern that they lead to delayed grafting or secondary graft malfunction, as well as the risk of interfering with graft-versus-lesion effect (GVL). In this article, we review the clinical data related to the use of JAK inhibitors in the peri-transplantation period of hematopoietic stem cell transplantation for primary myelofibrosis and discuss their efficacy and safety.

## JAK/STAT signaling pathway and JAK inhibitors

Janus kinase (JAK) is a non-receptor tyrosine kinase. It comes in forms of JAK 1, JAK 2, JAK 3, and TYK 2 in mammals. JAK 3 is expressed predominantly in hematopoietic cells, and the others are expressed in a wide range of tissues [7, 8]. Many cytokines and growth factors have been found to signal through the JAK/STAT pathway, including interleukins (ILs), erythropoietin (EP0), thrombopoietin (TPO), granulocyte/macrophage colony-stimulating factor (GM-CSF), platelet-derived factor (PDGF), epidermal growth factor (EGF), and interferons (IFNs). Each JAK member can be activated by a variety of cytokines, and together they constitute a family of molecules that rapidly transmit signals from the cell membrane to the nucleus without the need for a second messenger, and are involved in the regulation of proliferation, differentiation, migration, and apoptosis in a wide range of cells [9–12]. Persistent activation of the JAK/STAT signaling pathway is associated with hematologic malignancies, especially myeloproliferative neoplasms, and is considered an attractive molecular target. Increasingly, studies have shown that blocking JAK phosphorylation inhibits cytokine-induced immune activation and inflammatory responses, and that JAK inhibitors have significant immunosuppressive activity on dendritic cells, natural killer cells, helper T cells and regulatory T cells of the immune system [13, 14]. GVHD currently remains the leading cause of non-recurrent mortality (NRM) in allo-HSCT. GVHD is a complex pathological process involving numerous cytokines at each step of the way, from tissue inflammation caused by pretreatment therapy, through T-cell activation due to the interaction between antigen-presenting cells (APCs) and allogeneic T cells, to tissue damages by the migration of immune cells [15]. Significant efficacy has also been observed with JAK inhibitors, represented by ruxolitinib (RUX), for the treatment of steroid-resistant GVHD.

A variety of JAK inhibitors are currently on the market or under clinical studies (see Table 1), and these drugs have different target selectivity [10, 16]. Tofacitinib, targeting JAK 1 and JAK 3, is the world’s first JAK inhibitor approved for clinical use [17, 18], and has shown encouraging efficacy in a variety of autoimmune diseases such as rheumatoid arthritis, ankylosing spondylitis, ulcerative colitis, and polyarticular juvenile idiopathic arthritis [7, 8, 19]. Ruxolitinib (RUX) is a JAK1/2 tyrosine kinase inhibitor and it also partially inhibits TYK2. JAK1 and Tyk2 regulate pro-inflammatory cytokine signaling. JAK2 is involved in pro-erythropoietin and thrombopoietin signaling [7, 8]. RUX is currently approved for the treatment of PMF, PV, and graft-versus-host disease, with the most clinical experience accumulated with the drug in these diseases [20–22]. However, off-target effects often cause adverse reactions such as hemocytopenia and infections due to suboptimal target selectivity of RUX [22]. Currently, many emerging JAK inhibitors are entering clinical trials with the aim of reducing therapeutic toxicity and maximizing clinical efficacy.

**Table 1:** JAK inhibitors and their approved indications

JAK inhibitors	Selectivity	Approved indications
Tofacinitib	JAK3, JAK1, JAK2	rheumatoid arthritis, ulcerative colitis, juvenile idiopathic arthritis, and psoriatic arthritis
Ruxolitinib	JAK1 and JAK2	
Baricitinib	JAK1 and JAK2	Rheumatoid arthritis, atopic dermatitis, alopecia areata and COVID-19
Momelitinib	JAK1 and JAK2	/
Brepocitinib	JAK1 and Tyk2	/
Itacitinib	JAK1	Lymphoma
Upadacitinib	JAK1, JAK2, JAK3	Rheumatoid arthritis, ankylosing spondylitis, non-radiographic axial spondyloarthritis, atopic dermatitis and psoriatic arthritis
Filgotinib	JAK1	Rheumatoid arthritis
Abrocitinib	JAK1	Atopic dermatitis
Oclacitinib	JAK1	Myelofibrosis, rheumatoid arthritis, and psoriatic arthritis, atopic dermatitis
Fedratinib	JAK2	Myelofibrosis
Pacritinib	JAK2	High-risk myelofibrosis
Lestaurtinib	JAK2	/
Gandotinib	JAK2	/
Decernotinib	JAK3	Rheumatoid arthritis
Ritlecitinib	JAK3	Alopecia areata
Deucravacitinib	TYK2	Moderate to severe chronic plaque psoriasis
Delgocitinib	JAK1, JAK2, TYK2 and JAK3	Atopic dermatitis
Peficitinib	JAK3, JAK1, TYK2 and JAK2	Rheumatoid arthritis

## Timing of allogeneic hematopoietic stem cell transplantation for MF patients in the era of JAK inhibitors

In 2011, ruxolitinib (RUX) was approved in the United States for the treatment of medium-risk and high-risk MF, and was shown to be effective in improving patients’ symptoms, overall survival (OS), and quality of life [20, 23]. Analysis of 5-year data from the clinical studies COMFORT-I and COMFORT-II showed that RUX was associated with long-term control of splenic outgrowth [3, 24], and pooled analysis showed that reduction of splenic volume at week 24 of RUX treatment was associated with patients’ prolonged OS [25]. However, approximately 50% of the patients in the studies discontinued RUX within 3 years, and only 25% continued treatment after 5 years. The reasons for discontinuation included disease progression (20%), adverse events (20–25%) and unsatisfactory efficacy (5–10%). In a retrospective study, Kuykendall [26] et al. investigated 145 patients with MF treated with RUX outside of a clinical trial. Among them, 23 (16%) died during treatment, and 64 of the evaluable patients discontinued RUX, with the most common reasons for discontinuation being hemocytopenia (*n* = 24; 38%), anemia (*n* = 21; 33%), and thrombocytopenia (*n* = 9; 14%). Other reasons including receiving allo-HSCT (*n* = 10; 16%), lack of response (*n* = 9; 14%) or re-progression after an initial response (*n* = 7; 11%), progression to acute myeloid leukemia (*n* = 8; 13%) and development of treatment intolerance unrelated to hematopenia (*n* = 6; 10%). The prognosis for these patients was generally poor.

Ruxolitinib (RUX) does not cure myelofibrosis or reduce the risk of acute leukemic transformation, some patients even acquire new mutations to evolve clonally, so the only treatment with curative potential remains allo-HSCT [5, 6, 24]. MF patients undergoing allo-HSCT have a high morbidity and mortality from transplantation-related complications, especially graft failure and GVHD. According to data from the Center for International Blood and Marrow Transplantation Research (CIBMTR) on 289 MF patients undergoing allo-HSCT [27], the majority of the patients (*n* = 229) underwent myeloablative conditioning before transplantation, the 100-day transplant-related mortality was 18% for patients with HLA-matched relatives as the donors, compared to 35% and 19% for those with unrelated donors and those with HLA-partially matched relatives as donors, respectively. The 5-year overall survival rates for the three groups were 37%, 30% and 40%, respectively, with disease-free survival rates of 33%, 27% and 22%, and graft failure rates of 9%, 20% and 27%. Splenomegaly may lead to delayed engraftment and poor graft function, while splenectomy is associated with many perioperative complications. The use of RUX in the peri-transplantation period of MF has attracted significant attention due to its ability to shrink the spleen, improve myelofibrosis-related symptoms and physical status (e.g., night sweats and weight loss), and its potential to control the onset of GVHD [28, 29].

In the ruxolitinib era, the timing of allo-HSCT for MF patients is still unknown. Studies have shown that MF patients who respond to RUX have a better prognosis after transplantation than those who do not respond or lose response to RUX. In a retrospective study that included 551 patients with MF, 277 patients who were treated with RUX prior to HSCT and had a sustained splenic response to RUX had a 2-year cumulative relapse rate of 15.7%, compared to 8.1% in those who did not respond or lost response to RUX, while the event-free survival (EFS) was 68.9% and 49.9%, respectively, and 1-year non-relapse mortality (NRM) was 14.8% and 25.8% for the two groups of patients [30]. A large-scale retrospective study by Shanavas [31] et al. also reported better 2-year survival in patients who responded to RUX therapy (91% vs. 61%). The use of a pretransplant conditioning regimen containing Thiotepa for hematopoietic stem cell transplantation in recent years has been associated with improved tolerability of haploidentical grafts and significantly increased long-term survival after transplantation. Shouval et al. [32] reported an 80% progression-free survival (PFS) rate at 1 year in 12 patients using a combined regimen of Thiotepa, busulfan and fludarabine (TBF), whereas the EMBT [33] reported a 3-year OS of 55% in 187 MF patients who received the TBF regimen for transplantation. We recommend timely sequential transplantation rather than waiting for failure of JAK inhibitor therapy for patients with sustained splenic response during RUX therapy.

## Pre-transplantation ruxolitinib taper regimen and the risk of ruxolitinib discontinuation/withdrawal syndrome (RDS/RWS)

Considering that JAK2 is also a signaling pathway on which numerous hematopoietic growth factors are dependent, ruxolitinib (RUX) significantly affects hematopoiesis, leading to an inherent risk of delayed engraftment and graft malfunction after MF transplantation. Therefore, many centers have chosen to reduce or discontinue RUX prior to transplantation. RUX discontinuation may be followed by ruxolitinib discontinuation/withdrawal syndrome (RDS/RWS) [5, 34]due to an acute rebound of cytokine storm, which is characterized by rapid disease progression, worsening cytopenia, rapid increase in splenomegaly, and even hemodynamic instability, respiratory distress, shock, and possible cytokine release syndrome (CRS) [35]. In a survey of 251 patients whose RUX therapy was interrupted conducted by Palandri [36] et al., RDS occurred in 34 patients (13.5%) at a median time of 7 days (range 2–21 days) after RUX discontinuation, among whom 21 patients had mild RDS that was manifested as splenomegaly in 13 patients (61.8%), somatic symptoms (fever, weight loss, night sweats) in 2 patients, and other MF-related symptoms (malaise, pruritus, bone pain, abdominal discomfort) in 6 patients. Ten patients (29.4%) had moderate RDS, including 7 patients with splenomegaly and 3 with somatic symptoms. Three patients had severe RDS: one with splenic rupture; one with fever and respiratory distress; and one with severe ARDS, all of which appeared within 48 h after drug discontinuation, and rapidly improved after reintroduction of RUX.

There are a series of studies discussing strategies to reduce RUX prior to pretransplant conditioning. Shanavas [31] et al. reported that 66 patients with MF who were tapered off RUX prior to the conditioning, with 10 (15%) patients who developed new symptoms attributable to discontinuation, including severe events in 2 patients and mild to moderate events in 8 patients. Of the 21 patients with a discontinuation-to-conditioning interval of ≥ 6 days, 6 (29%) developed symptoms and 2 developed severe RDS, whereas of the 45 patients with an interval of < 6 days, only 3 (7%) patients developed mild-to-moderate symptoms. These suggest that RDS is more common in patients with longer intervals between the last administration of RUX and pretransplant conditioning. Jaekel [37] et al. reported that 14 patients, with a median RUX exposure of 3.11 months were tapered off RUX over 2 weeks prior to the start of pretransplant conditioning and the drug was completely discontinued when the conditioning was to start. Hanif et al. reported [38] that 8 patients at a steady-state dose of RUX of 20 mg BID for a median exposure of 180 days, were rapidly tapered off RUX from 6 days prior to pretreatment and discontinuation was achieved 24 h prior to pretreatment. Salit [39] reported that 28 patients with a median exposure of 7 months at the maximum tolerated dose of RUX had their dose reduced by 5 mg per day starting 9 days prior to transplantation, and the tapering was completed on day − 4 prior to stem cell infusion. RDS was not observed in any of these studies. Some studies also investigated whether the addition of steroids during RUX tapering reduced RDS. Gupta [40] reported 19 patients, who received pretransplant conditioning of reduced intensity, had been given RUX for at least 56 days before transplantation followed by tapering of RUX over 4 days and discontinuation 1–2 days before the conditioning. During the tapering, prednisone 30 mg /day was added, and no RDS was observed in any of the patients.

Abrupt discontinuation of ruxolitinib prior to pretreatment has been reported to be also feasible [41]. In a prospective study by Robin [42, 43] et al, a total of 2 events of febrile cardiogenic shock and 1 event of tumor lysis syndrome (TLS) with acute renal failure occurred in patients (*n* = 10) for whom ruxolitinib was tapered over 15 days. In the other group of patients (*n* = 42), RUX was abruptly discontinued before conditioning, and only 1 developed cardiogenic shock. In another study including 22 subjects [44], a direct discontinuation strategy was used and no patients developed RDS. In contrast to these previous studies that had small sample sizes, Palandri [36] et al. collected a real-world survey of MF patients from 22 sites that covered a total of 162 patients who had used the direct RUX discontinuation strategy and 89 patients who tapered off prior to discontinuation. The pattern of dose tapering varied widely among the sites and included dose reductions of 5 or 10 mg per day at various intervals, ranging from one dose reduction every 30 days to one dose reduction every 3 days. The median duration of the tapering was 14 days (range 3–60 days). No correlation was found between tapering regimen or the RUX dose at discontinuation and clinical/laboratory parameters. The 2015 European Primary Myelofibrosis Guidelines [1] recommend starting RUX ≥ 2 months before transplantation, followed by gradual adjustment to the maximum tolerated dose and slow tapering over 5–7 days before pretransplant conditioning to achieve complete discontinuation 1 day before the conditioning. Considering that RDS is associated with cytokine storm after RUX withdrawal, we suggest that the inflammatory cellular basis of RDS can be effectively eliminated if drug withdrawal is immediately followed by pretreatment chemotherapy and immunosuppressive therapy. The rate of Ruxolitinib tapering may not be a critical factor, and initiating pretransplant conditioning promptly following discontinuation of the medication could potentially mitigate the risk of RDS.

## Efficacy and safety of continued use of ruxolitinib after transplantation

Many studies suggest that continued use of ruxolitinib (RUX) after transplantation may reduce the risk of relapse and GVHD by reducing disease burden and inhibiting pro-inflammatory cytokines. Several clinical studies are focus on the efficacy and safety of using RUX consistently until stem cell infusion, hematopoietic recovery or 100 days after transplantation. Joanne E. Davis [45] et al. observed in a preclinical mouse model that ruxolitinib-treated mice had reduced NK and CD8 + T-cells, reduced acute GVHD incidence without affecting the stem cell engrafting, prolonged survival of experimental animals, and a significant GVT effect. In a retrospective cohort study by Pu [46] et al., patients were divided into three cohorts, A (*n* = 3) with no prior treatment with RUX, B (n-9) with only pre-transplant RUX, and C (*n* = 4) with RUX treatment before and after transplantation. Cohort C had a greater reduction in splenic dimensions and a faster engrafting rate than cohorts A and B. Up to the last follow-up, all patients were alive. In cohort A, two of the three patients had stable disease (SD) and one had disease progression (PD). In cohort B, the numbers of patients achieving CR, PR, and SD were 2, 2 and 5, respectively, and all four patients in cohort C reached CR at a median of 11.5 months. In terms of GVHD, cohort C showed a lower incidence of aGVHD (0/4) compared to cohort A (3/3) or B (4/9). In the report by Kröger et al., [47] 12 MF patients who continued RUX (2–5 mg per day) during transplantation until stable engraftment, with median exposure of 163 days to RUX prior to transplantation, all showed improvement in spleen size and physical symptoms, and all achieved successful engraftment. The median time to leukocyte engraftment was 12 days (range 11–18). After a median of 40 days, 11 patients achieved complete donor chimerism, and after a median of 32 days, molecular clearance of potential driver mutations (JAK2V617F, CALR, or MPL) was noted in 10 patients. Before pretransplant conditioning, patients were given RUX 5 mg twice daily, which was tapered to 5 mg once a day on day + 20 post-transplantation and discontinued on day + 28 post-transplantation. Due to hypocytosis, two patients discontinued RUX early on day+17 and day+18 post-transplantation respectively. One patient developed unexplained fever after discontinuation of the drug, no other symptoms of RDS were observed. One case each of grade 1 and grade 2 acute GVHD occurred during treatment, but the incidence of cytomegalovirus (CMV) reactivation was high with five events (41%).

In a prospective phase I clinical trial organized by Haris Ali [34] et al., RUX was administered twice a day at 2 dose levels of 5 and 10 mg from day 23 before transplantation to day 130 after transplantation. 6 and 12 patients were enrolled in the two dose groups respectively. Granulocyte engraftment was achieved at a median of 19 days (range, 13–23) and a median of 16 days (range, 12–22) respectively, and platelet engraftment was achieved at a median of 20 days (range, 19–42) and a median of 28 days (range, 13–119) in the two groups, with four patients failing to achieve platelet transfusion independence. Blood or bone marrow chimerism was 100% in all patients at 1 year. The cumulative incidence of grades 2–4 and grades 3–4 acute GVHD in the whole cohort was 45% and 17% respectively, the cumulative incidence of 1-year chronic GVHD was 42%, and 1-year overall and progression-free survival rates were 77% and 71%, respectively. Morozova [48] et al. conducted a prospective study evaluating RUX in combination with post-transplantation cyclophosphamide (PTCY) for a regimen to prevent calcineurin phosphatase inhibitor-free GVHD in a MF population. The GVHD prophylaxis regimen consisted of PTCY (50 mg/kg) on days + 3 and + 4 and 15 mg of RUX daily from days + 5 to + 100. The study documented initial engraftment in 17 patients, among whom 2 patients died before and 1 patient died after engraftment. The median time to neutrophil engraftment was 27 days (18–44), that to platelet implantation was 38 days (15–219), and that to transfusion independence was 59 days (20–540). Severe graft dysfunction (SPGF) was observed in a total of 55% (*n* = 11) of the patients, which was resolved in 8 patients with dose reduction of RUX. One patient experienced primary graft failure and underwent a second HSCT, and the patient is currently living in remission. In this study, the incidence of grades 2–4 aGVHD was 25% and the overall incidence of chronic GVHD was 40%, all of which was mild to moderate cGVHD, only 2 patients required systemic steroid therapy. One patient experienced recurrence 665 days after transplantation. The 2-year NRM, OS, and EFS were 15%, 85%, and 72%, respectively. These findings suggest that the use of RUX in the peri-transplantation period is relatively safe and feasible [4].

## Application of other JAK inhibitors

Despite the ample experience with RUX in the peri-transplantation period for MF, the target selectivity of the drug is still unsatisfactory. In addition to targeting JAK1/JAK2, it has significant affinity for JAK3 and Tyk2. Since these four JAK family members control approximately 40 cytokine receptor signaling pathways, RUX can affect multiple cytokine signaling pathways, leading to off-target effects [49]. Many patients are forced to reduce or discontinue RUX due to dose-dependent cytopenia, limiting its therapeutic effect as well as increasing resistance. An increasing number of JAK inhibitors are now entering clinical studies, potentially leading to new options for MF patients.

Fedratinib, a JAK2 inhibitor that is more selective than RUX and also inhibits FLT3 and BET, with a longer effective half-life period, induces a strong splenic response. It is the second JAK inhibitor approved by the FDA for the treatment of medium- to-high-risk MF [50–53]. In the JAKARTA trial, patients with medium- to-high-risk MF with no prior exposure to a JAK inhibitors were divided into three groups: fedratinib at 400 mg/day, fedratinib at 500 mg/day, and placebo. In these three groups, 36%, 40%, and 1% of patients, respectively, achieved a splenic response (≥ 35% reduction in splenic volume from baseline) at week 24, and 36%, 34% and 7%, respectively, of patients achieved symptomatic improvement [54, 55]. In the JAKARTA2 trial in MF patients previously treated with RUX, 55% of the patients achieved a splenic response. Common adverse events with this drug are anemia, thrombocytopenia and gastrointestinal symptoms [56]. However, a case of suspected Wernicke’s encephalopathy has been reported (Wernicke’s encephalopathy is a rare neurodegenerative disorder caused by thiamine deficiency, with the classic triad of oculomotor paralysis, ataxia, and psychiatric disorders).

Pacritinib, which is specific for JAK2, FLT3 and IRAK1 but does not affect JAK1, has demonstrated clinical activity in MF, and due to its mild myelosuppression, it was approved in the U.S. in 2022 for the treatment of MF patients with platelet counts less than 50 × 109/L [57–59]. In the dose-finding PAC203 trial, [60] pacritinib 200 mg BID was shown to maximize spleen volume reduction and symptom improvement.

Momelotinib, a JAK1, JAK2, and ACVR1 inhibitor, inhibits ACVR1-mediated ferroportin production, increases serum iron utilization and stimulates red blood cell production [61]. Three phase 3 studies (SIMPLIFY-1, SIMPLIFY-2 and MOMENTUN) demonstrated its sustained symptomatic improvement and favorable safety. It is indicated that the efficacy of the treatment for MF patients suffering from symptoms of anemia with the requirement for red blood cell transfusion is good, with  70% of patients resolved in at least 12 weeks [62, 63]. However, a high incidence of peripheral neuropathy (30-50%) has also been reported with the drug.

Jaktinib, a deuterated form of Momelotinib, was shown in a phase 3 study (NCT04617028) to reduce spleen volume by ≥ 35% (SVR35) in 72.3% of the patients at Week 24 at a dose of 100 mg BID. It is a promising treatment option for patients with MF who develop refractory or recurrent disease after RUX therapy [64–66, 56, 67].

Gandotinib is an effective inhibitor of JAK2 activity with enhanced potency against the JAK2V617F mutation and has not exhibited the hematologic or infectious toxicity reported with RUX. Nor has it exhibited the neurotoxicity or serious safety concerns seen with other JAK inhibitors [68]. A phase I study suggested improvement in symptom assessment scale and splenic size following gandotinib treatment, where the maximum tolerated dose was 120 mg daily, and 29% of the MF patients achieved a best response of clinical improvement [69].

There is still a lack of data on the pre-transplantation use of fedratinib, pacritinib, momelotinib, jaktinib, and gandotinib, but their efficacy in MF suggests that they may also have a broad potential in pre-transplantation bridging therapy for MF.

## Conclusion

In summary, JAK inhibitors, represented by ruxolitinib (RUX), can shrink the spleen and improve myelofibrosis-related symptoms and physical status, with the potential to control the development of GVHD. Their use in the peri-transplantation period of MF may improve the success rate of allo-HSCT in patients with MF, with acceptable concomitant adverse effects. At present the post-HCT impact of peri-implantation use of RUX or other JAK inhibitors awaits support from larger prospective randomized trials.

## Data Availability

All data are available upon request.
